# Nadir PSA level and time to nadir PSA are prognostic factors in patients with metastatic prostate cancer

**DOI:** 10.1186/1471-2490-14-33

**Published:** 2014-04-29

**Authors:** Atsushi Tomioka, Nobumichi Tanaka, Motokiyo Yoshikawa, Makito Miyake, Satoshi Anai, Yoshitomo Chihara, Eijiro Okajima, Akihide Hirayama, Yoshihiko Hirao, Kiyohide Fujimoto

**Affiliations:** 1 Department of Urology, Nara Medical University, 840 Shijo-cho, Kashihara, Nara 634-8522, Japan

**Keywords:** Prostate cancer, Metastasis, Risk factors

## Abstract

**Background:**

Primary androgen deprivation therapy (PADT) is the most effective systemic therapy for patients with metastatic prostate cancer. Nevertheless, once PSA progression develops, the prognosis is serious and mortal. We sought to identify factors that predicted the prognosis in a series of patients with metastatic prostate cancer.

**Methods:**

Two-hundred eighty-six metastatic prostate cancer patients who received PADT from 1998 to 2005 in Nara Uro-Oncology Research Group were enrolled. The log-rank test and Cox’s proportional hazards model were used to determine the predictive factors for prognosis; rate of castration-resistant prostate cancer (CRPC) and overall survival.

**Results:**

The median age, follow-up period and PSA level at diagnosis were 73 years, 47 months and 174 ng/mL, respectively. The 5-year overall survival rate was 63.0%. The multivariable analysis showed that Gleason score (Hazard ratio [HR]:1.362; 95% confidence interval [C.I.], 1.023-1.813), nadir PSA (HR:6.332; 95% C.I., 4.006-9.861) and time from PADT to nadir (HR:4.408; 95% C.I., 3.099-6.271) were independent prognostic factors of the incidence of CRPC. The independent parameters in the multivariate analysis that predicted overall survival were nadir PSA (HR:5.221; 95% C.I., 2.757-9.889) and time from PADT to nadir (HR:4.008; 95% C.I., 2.137-7.517).

**Conclusions:**

Nadir PSA and time from PADT to nadir were factors that affect both CRPC and overall survival in a cohort of patients with metastatic prostate cancer. Lower nadir PSA level and longer time from PADT to nadir were good for survival and progression.

## Background

Prostate cancer is the fourth most commonly diagnosed cancer and the ninth leading cause of cancer deaths among males in Japan [1]. At the moment, Japanese prostate cancer patients show higher risk characteristics compared with the patients in the United States, and the proportion of metastatic patients is still high [2,3]. Whereas primary androgen deprivation therapy (PADT) is standard for metastatic prostate cancer, most patients progress to castration-resistant prostate cancer (CRPC) at various intervals after PADT. Prostate specific antigen (PSA) is a biomarker for diagnosis, risk classification, and monitoring of the disease. Most patients will experience a substantial decline in PSA levels, and their PSA levels may remain low or undetectable for years. Nevertheless, CRPC occurs frequently [4]. CRPC follows an androgen-independent state, which leads to widespread metastases. PSA progression in advanced prostate cancer indicates clinical progression in patients treated with PADT within a median of 6 months [5]. The substantial variability in the clinical course of metastatic prostate cancer has led to the evaluation of a number of prognostic factors with respect to their roles in determining the treatment strategy and ability to predict the response to therapy. In most clinical trials of prostate cancer, an improvement of 50% in the serum PSA is used as a marker of response [6,7]. The criteria for disease progression when using changes in PSA is defined by the Prostate Cancer Clinical Trials Working Group (PCWG2) [6] and the Prostate-Specific Antigen Working Group (PSAWG) [7], and has been validated by the data-sets from two large Southwest Oncology Group Trials (SWOG 9346 and 9916) [8]. We endeavored to identify risk factors for prognosis in our series of patients with hormone naïve metastatic prostate cancer.

## Methods

This study retrospectively evaluated 286 Japanese patients with metastatic prostate cancer who received PADT following diagnosis in the Nara Uro-Oncology Research Group (NUORG) between January 1998 and December 2005. The diagnosis was based on prostate biopsy. Abdominal computed tomography and/or bone scans were used in all cases with suspected metastases. All patients were treated with PADT using a luteinizing hormone-releasing hormone (LH-RH) agonist, surgical castration, anti-androgen monotherapy or combined androgen blockade (CAB).

Follow-up data were retrieved from the hospital medical records. Patients were followed every month for the first 3 months and every 3 months thereafter. The nadir PSA level was defined as the lowest PSA level after PADT. CRPC was defined as the first day when the PSA was increased for three consecutive times or when clear clinical radiological evidence of progressive disease was shown. This study analyzed the incidences of progression times to CRPC after the initiation of PADT and overall survival time. The progression rate to CRPC and overall survival rate were estimated and both univariate and multivariate analyses were carried out to determine the prognostic value of age (≤75 years vs. ≥76 years), TNM classification (UICC 2002 [9]), pathological Gleason score (6–8 vs. 9–10), PSA level at diagnosis (<100 ng/mL vs. 100–500 ng/mL vs. ≥500 ng/mL), nadir PSA level (<0.2 ng/mL vs. 0.2-4 ng/mL vs. ≥4 ng/mL), time from PADT to nadir (≥12 months vs. 6–12 months vs. <6 months) and time from PADT to CRPC (No occurrence of CRPC vs. ≥12 months vs. 6–12 months vs. <6 months).

Statistical analyses were carried out using the SPSS software package (SPSS Inc., Chicago, Illinois, version 17.0) and p < 0.05 was considered to be statistically significant. Overall survival was estimated by the Kaplan-Meier method. The log rank test was used to assess differences between groups. The Cox proportional hazards regression model was performed to analyze independent predictors of CRPC and overall survival. Only the variables that were found to be significant in the univariate analyses (p < 0.05) were entered into the multivariate analysis to determine the most significant factor for predicting disease outcome.

The Medical Ethics Committee of Nara Medical University approved this retrospective study, and it was exempted to obtain informed consent from the patients in consideration of the aim and methods of this study.

## Results

The characteristics of the patients are shown in Table 1. The median age and PSA at diagnosis were 73 years (range 50 to 92) and 174 ng/mL (range 5.7 to 21864), respectively. The median follow-up was 47 months (range 2 to 128) and the treatment of PADT was CAB in 92.0% of the patients. There were no differences in the progression time to CRPC and overall survival between PADTs (LH-RH agonist, surgical castration, anti-androgen monotherapy or CAB) (Data are not shown). Most patients initially responded to PADT. The median nadir PSA level was 0.3 ng/mL (range 0.001 to 650) and 42.7% of patients reached a nadir PSA <0.2 ng/mL. The median time from PADT to nadir was 9.45 months (range 1 to 64). 72.4% of patients progressed to CRPC and the median time to progression after the initiation of treatment was 13 months (range 1 to 97). The 5-year overall survival rate was 63.0%, the median survival time from CRPC was 45 months, and 35.7% of patients died during the follow-up period, of which cancer deaths and other cause deaths were 27.6% and 8.0%, respectively.

**Table 1:** Characteristics of patients and the results of the log-rank test of prognostic factors

		**Median (range)**	**No. of patients**	**No. of CRPC**	**Median progression time (months)**		**No. of deaths**	**Median survival time (months)**	
Total			286	207	19		102	113	
Age		73 (50–91)							
	≤75		179	132	19		69	85	
	≥76		107	75	18	n.s.	33	none	n.s.
T	T1		4	2	57	*(T2 vs. T3, T4)	0	all alive	
	T2		31	18	37		9	111	
	T3		162	114	19		60	83	
	T4		89	73	14		33	77	n.s.
N	N0		164	116	19		59	79	
	N1		122	91	19	n.s.	43	99	n.s.
M	M0		50	31	26		9	99	
	M1		236	176	18	n.s.	93	83	n.s.
Gleason score	6-8		181	83	21	*	63	115	
	9-10		105	124	14		39	85	n.s.
PSA at diagnosis		174 (5.7-21864)							
	<100		105	64	23	*(<100 vs. ≥500)	31	85	
	100-500		97	73	16		36	113	
	≥500		84	70	15		35	none	n.s.
Nadir PSA level		0.3 (0.001-650)							
	<0.2		122	71	38	**	26	115	***
	0.2-4		112	86	13		44	62	
	≥4		52	50	8		32	25	
Time from PADT to Nadir		9.45 (1–64)							
	≥12		114	74	35	**	23	113	***
	6-12		83	62	13		39	79	
	<6		89	71	7		40	28	
Time from PADT to CRPC		13 (1–97)							
	No CRPC		79				14	none	
	≥12		112				32	113	***
	6-12		63				34	35	
	<6		32				22	21	

*P<0.005, **all P<0.001, ***P<0.001 without No CRPC vs. ≥12

PADT: primary androgen deprivation therapy.

CRPC: castration resistant prostate cancer.

T stage (T2 vs. T3, T4), Gleason score (6–8 vs. 9–10), PSA at diagnosis (<100 ng/mL vs. ≥500 ng/mL), nadir PSA (<0.2 ng/mL vs. 0.2-4 ng/mL) and time from PADT to nadir (≥12 months vs. 6–12 months vs. <6 months) were significantly associated with the progression time to CRPC (Table 1). A lower nadir PSA level and a longer time from PADT to nadir were associated with a lower proportion of patients with CRPC progression. The median progression time to CRPC of the patients with nadir PSA <0.2 ng/mL, 0.2-4 ng/mL and ≥4 ng/mL were 38 months, 13 months and 8 months, respectively (p < 0.005). The median progression time to CRPC of the patients whose time from PADT to nadir were ≥12 months, 6-12 months and <6 months, were 35 months, 13 months and 7 months, respectively (p < 0.005). The median nadir PSA level of the patients with time from PADT to nadir ≥12 months, 6–12 months and <6 months, were 0.1 ng/mL, 0.3 ng/mL and 1.18 ng/mL, respectively (p < 0.05: ≥12 months vs. <6 months). The univariate analysis showed that the Gleason score, PSA at diagnosis, nadir PSA and time from PADT to nadir were associated with progression to CRPC (Table 2). The multivariate analysis showed that Gleason score, nadir PSA and time from PADT to nadir were significant independent factors.

**Table 2:** Hazards ratio estimate and confidence intervals from proportional hazards modeling of progression to CRPC

		**Univariate analysis**			**Multivariate analysis**		
		**Hazard ratio**	**95% C.I.**	***P *** **value**	**Hazard ratio**	**95% C.I.**	***P *** **value**
Age		0.993	0.975-1.011	0.425	0.990	0.972-1.007	0.248
T	T1	1					
	T2	1.359	0.315-5.867	0.681			
	T3	2.142	0.528-8.691	0.286			
	T4	3.336	0.816-13.647	0.094			
Gleason score	6-8	1			1		
	9-10	1.518	1.148-2.009	0.003	1.362	1.023-1.813	0.034
PSA at diagnosis	<100	1			1		
	100-500	1.374	0.981-1.923	0.064	0.990	0.691-1.417	0.955
	≥500	1.616	1.149-2.271	0.006	1.099	0.682-1.492	0.965
Nadir PSA level	<0.2	1			1		
	0.2-4	2.778	2.011-3.836	<0.001	2.794	1.984-3.936	<0.001
	≥4	6.339	4.315-9.313	<0.001	6.332	4.066-9.861	<0.001
Time from PADT to nadir	≥12	1			1		
	6-12	2.091	1.480-2.953	<0.001	2.245	1.559-3.232	<0.001
	<6	4.131	2.956-5.774	<0.001	4.408	3.099-6.271	<0.001

95% CI.: 95% confidence interval.

PADT: primary androgen deprivation therapy.

CRPC: castration resistant prostate cancer.

The log-rank test showed that overall survival was correlated with nadir PSA (<0.2 ng/mL vs. 0.2-4 ng/mL vs. ≥4 ng/mL), time from PADT to nadir (≥12 months vs. 6–12 months vs. <6 months) and time from PADT to CRPC (≥12 months vs. 6–12 months vs. <6 months) (Table 1). The multivariate analysis showed that nadir PSA and time from PADT to nadir were independent factors associated with overall survival (Table 3).

**Table 3:** Hazards ratio estimate and confidence intervals from proportional hazards modeling of overall survival

		**Univariate analysis**			**Multivariate analysis**		
		**Hazard ratio**	**95% C.I.**	***P *** **value**	**Hazard ratio**	**95% C.I.**	***P *** **value**
Age		0.987	0.962-1.013	0.324	0.994	0.967-1.021	0.647
Nadir PSA level	<0.2	1			1		
	0.2-4	3.038	1.852-4.982	<0.001	2.329	1.342-4.040	0.003
	≥4	7	4.109-11.925	<0.001	5.221	2.757-9.889	<0.001
Time from PADT to nadir	≥12	1			1		
	6-12	2.544	1.473-4.393	0.001	1.483	0.789-2.789	0.221
	<6	6.918	4.174-11.467	<0.001	4.008	2.137-7.517	<0.001
Time from PADT to CRPC	No CRPC	1			1		
	≥12	0.963	0.509-1.822	0.907	0.718	0.367-1.407	0.335
	6-12	4.323	2.310-8.089	<0.001	1.419	0.664-3.031	0.367
	<6	9.021	4.570-17.808	<0.001	1.712	0.731-4.005	0.215

95% CI.: 95% confidence interval.

PADT: primary androgen deprivation therapy.

CRPC: castration resistant prostate cancer.

## Discussion

A PSA test provides useful information, not only for the screening of prostate cancer but also for monitoring following treatment. Moreover, PSA monitoring before and after PADT are useful to evaluate the response to treatment in patients with prostate cancer. PSA concentrations decrease by 80% in approximately 80% of patients in the first month following PADT [10], and normalize in 95% of cases within 3–6 months [11]. Rising PSA after the nadir value under PADT represents the first objective sign of CRPC. PSA recurrence usually predates clinical progression of metastatic prostatic cancer after PADT [6,12]. Most of the patients in the current series initially responded to PADT; 43.2% of patients reached a nadir PSA <0.2 ng/mL, 72.4% had progression to CRPC and the median time from PADT to CRPC was 13 months.

We sought to identify risk factors for the prognosis of a series of patients with metastatic prostate cancer before or during PADT. We believe that it could be useful to decide on the best treatment strategy to predict which patients are more likely to develop early progression to CRPC. There are many views about the prognostic value of the PSA level at diagnosis. Several PSA-related parameters have been reported, including PSA at diagnosis, pattern of PSA decrease after treatment, time to nadir PSA and percentage of PSA decrease. Some authors found that the PSA level at diagnosis did not predict the time to progression [5,13]. However, others have proposed that PSA at diagnosis predicts the disease response to androgen suppression [14-16]. Early normalization of PSA delays the time to progression, and in combination with the Gleason score, PSA is an important prognostic factor to predict the efficacy of the therapy [17]. The decline of PSA to <4 ng/mL after the initiation of PADT within 3 months is thought to be more important than the Gleason score in determining the time to progression [17].

Nadir PSA after PADT is usually evaluated in relation to progression to CRPC [13,16,18-21]. The ability to achieve an undetectable PSA level as nadir is the most significant predictor of the time to CRPC for metastatic and advanced prostatic cancer and the time to nadir PSA is significantly and positively correlated with the PSA progression-free survival [13]. Failure to attain a nadir PSA of <1 ng/mL after treatment predicts early progression to CRPC [20,21]. In the current series, the median nadir PSA level was 0.3 ng/mL. We classify the nadir PSA level into <0.2 ng/mL, 0.2-4 ng/mL and ≥4 ng/mL, because PSA level <0.2 ng/mL is an undetectable PSA and the normal PSA value is less than 4 ng/mL. Then, nadir PSA (<0.2 ng/mL vs. 0.2-4 ng/mL vs. ≥4 ng/mL) was a significant independent factor that predicted the progression to CRPC after PADT.

Nadir PSA levels after PADT are usually evaluated in relationship to the overall survival time. A nadir PSA of >2 ng/mL predicts poorer overall survival [21]. By using the data of a randomized phase 3 trial, a PSA level of ≤4 ng/mL after 7 months of PADT was a strong independent predictor of improvement survival in metastatic hormone-sensitive prostate cancer [22]. Time to nadir < 6 months, Gleason score >7 and nadir PSA ≥0.2 ng/mL independently predicted shorter overall survival in patients with metastatic hormone-sensitive prostate cancer [18], which included patients who underwent definitive initial local therapy (radical prostatectomy or radiotherapy) and did not showed the factor of time to CRPC after initiation of PADT. Nadir PSA <0.2 ng/mL and longer time to nadir (>9 months) during PADT are the most important early predictors for survival in prostate cancer patients with bone metastasis [23]. The current study included the metastatic prostate cancer patients treated only with PADT, also predicts the factor of progression to CRPC and showed a nadir PSA (<0.2 ng/mL vs. 0.2-4 ng/mL vs. ≥4 ng/mL) and the time from PADT to nadir (≥12 months vs. 6–12 months vs. <6 months) were independent factors for overall survival. Nadir PSA and time from PADT to nadir are important factors associated with both CRPC and overall survival. This indicates that a simple measurement of PSA was strongly associated with time to CRPC and overall survival in metastatic CRPC, and the optimal cut-off point of the nadir PSA and the time from PADT to nadir predicts a short or long response of PADT.

Longer time to reach nadir PSA was associated with a lower nadir PSA level and this may simply indicate continued androgen sensitivity. The mechanisms responsible for the association of shorter time to nadir with a worse prognosis are not clear. A rapid decrease in the PSA level may be related to a transcriptional effect of PADT on PSA progression rather than prostate cancer cell death [20]. A rapid decrease in the PSA level after PADT may be due to ablation of the androgen receptor function, and quick suppression of androgen/androgen receptor during PADT may have a negative effect on disease progression, because the androgen receptor can act as a tumor suppressor for prostate cancer [24]. Another possibility is that a rapid removal of hormone-sensitive prostate cancer cells may induce an environment that allows the growth of hormone-resistant prostate cancer cells [23].

There are limitations to the current study. Firstly, some important factors, such as lactate dehydrogenase, alkaline phosphatase, albumin, testosterone, hemoglobin and performance status were not included in this analysis, because they were not routinely measured in the patients. Secondly, we only reported the outcomes of PADT but not evaluated the following results of second-line treatment such as chemotherapy. Thirdly, there may be interobserver variation of the Gleason score between general pathologists and uropathologists.

## Conclusions

PSA progression and PSA decline provide clinically meaningful information early during the treatment course of patients with metastatic prostate cancer who are unlikely to benefit from standard PADT long before they develop CRPC. Further prospective data and external validation of independent datasets are needed to confirm these findings.

Lower nadir PSA value and longer time to nadir PSA after the initiation of PADT correlate with lower progression to CRPC and higher overall survival.

## Abbreviations

PADT: Primary androgen deprivation therapy; CRPC: Castration-resistant prostate cancer; PSA: Prostate specific antigen; LH-RH: Hormone-releasing hormone; CAB: Combined androgen blockade.

## Competing interests

The authors declare that they have no competing interests.

## Authors’ contributions

AT contributed to analysis and interpretation of data and was involved in drafting the manuscript. TN contributed to conception and helped to draft the manuscript. MY, MM, SA and YC contributed to acquisition of data. EO and AH contributed to acquisition of data and helped to draft the manuscript. YH and KF conceived and supervised the study, helped to draft the manuscript and was involved in revising it critically for important intellectual content. All authors read and approved the final manuscript.

## Pre-publication history

The pre-publication history for this paper can be accessed here:

http://www.biomedcentral.com/1471-2490/14/33/prepub
